# Design My Music Instrument: A Project-Based Science, Technology, Engineering, Arts, and Mathematics Program on The Development of Creativity

**DOI:** 10.3389/fpsyg.2021.763948

**Published:** 2022-01-05

**Authors:** Li Cheng, Meiling Wang, Yanru Chen, Weihua Niu, Mengfei Hong, Yuhong Zhu

**Affiliations:** ^1^Faculty of Education, Beijing Normal University, Beijing, China; ^2^Developmental and Educational Research Center for Children's Creativity, FE, Beijing Normal University, Beijing, China; ^3^Department of Psychology, Pace University, New York, NY, United States; ^4^Hangzhou Caihe No.2 Primary School Education Group, Zhejiang, China; ^5^Department of Physics, Hangzhou Normal University, Zhejiang, China

**Keywords:** STEAM curriculum, project-based learning, creativity, learning by doing, multi-method approach

## Abstract

Creativity is an essential factor in ensuring the sustainable development of a society. Improving students’ creativity has gained much attention in education, especially in Science, Technology, Engineering, Arts, and Mathematics (STEAM) education. In a quasi-experimental design, this study examines the effectiveness of a project-based STEAM program on the development of creativity in Chinese elementary school science education. We selected two fourth-graders classes. One received a project-based STEAM program (the experimental group, *n* = 33), and the other received a conventional science teaching (the control group, *n* = 33) over 6 weeks. Students’ creativity was assessed before and after the intervention using a multi-method approach, including a test of divergent thinking, a story completion through the Consensus Assessment Technique (CAT), a creative self-efficacy (CSE) measure, and a group-based creative project. Moreover, all students received a test of their science knowledge after the intervention. The results showed that compared with the control group, the creativity of the experimental group students improved significantly for 6 weeks at both individual and group level, even though their knowledge in science were comparable. This result confirmed the effectiveness of a project-based STEAM educational program improving elementary school students’ creativity. Implications are discussed.

## Introduction

Creativity has become an increasingly important factor in ensuring the sustainable development of a society and is one of the essential skills in the 21st century. The definition of creativity has been evolved. Guilford (1950) referred to creativity as one of many aspects of intelligence and believed it included two thinking processes: divergent and convergent thinking. To Gilford, divergent thinking stimulates individuals to generate new ideas and make creative products novel. In contrast, convergent thinking is conducive to the individual’s idea of adapting to the environment and increasing the applicability of new products. Later, Torrance (1963) proposed creativity as creative problem-solving, and creative thinking was viewed as a particular method or form to solve problems. Toward the end of the 20th century, there is a consensus to the definition of creativity among creativity researches that creativity is a person’s ability to produce something that is deemed as novel and appropriate by experts of a field (Amabile, 1996; Csikszentmihalyi, 1997; Lubart, 1999; Kaufman and Beghetto, 2009).

Many theories have been proposed to explore the nature and contributing factors for creativity *via* different approaches, such as psychometric, cognitive, developmental, and social approaches. Most scholars took the confluence approach in which creativity is viewed as an ability to be influenced by multi-factors. Besides examining different elements that contribute to the development of creativity, some scholars focus on exploring creativity at different levels. Many scholars took a dichotomies view and concentrate on examining creativity at either eminent level, such as studying creative genius and their work (e.g., Simonton, 1994), or ordinary level such as everyday creativity (e.g., Torrance, 1969). These studies typically referred to creativity as Big-C or little-C (Richards, 1990; Kaufman and Beghetto, 2009). In advancing this approach, Kaufman and Beghetto (2009) proposed a new model, which they called the Four-C model of creativity. According to this model, creativity can be manifested in four different levels, namely, the Big-C, referring to the creative genius who brought a significant breakthrough in a field, the Pro-C, referring to the type of creativity that is important to an area but not at the level of a substantial breakthrough. The third level of creativity is called little-c, also referred to as everyday creativity; the type of creativity exhibited by ordinary people, yet the level of creativity is still needed to be recognized by experts in a given field. Lastly, there is a type of creativity that is only significant to individuals without fully been recognized by others. Kaufman and Beghetto called it mini-c.

Some scholars have suggested using a multi-method approach to capture creativity (Cropley, 2000). The multi-approach of studying creativity at a multilevel indicates that no single method can fully capture creativity. In other words, creativity has to be measured *via* a multi-method approach. The most common measurements to creativity include divergent thinking, such as the Torrance Test of Creative Thinking (TTCT), product-orientated measure *via* consensus assessment technique (CAT), various self-report measurements on creative personality, activities, thinking styles, and creative self-efficacy (CSE), as well as ratings from others, such as teachers, parents, experts, and peers. These methods measure the level of little-c and mini-c.

Another critical question in creativity is how to nurture creativity. It is especially important in education (Scott et al., 2004; Sannomiya and Yamaguchi, 2016). One approach to fostering creativity is by promoting intrinsic motivation (Amabile, 1988, 1996). Another approach is through innovative teaching (Baer, 1996). Many studies have been conducted to actively explore effective educational models to improve students’ creative abilities. Among them, the approach of Science, Technology, Engineering, Arts, and Mathematics (STEAM) education provides a comprehensive and practical method to develop creativity and has gained worldwide popularity.

### STEM and STEAM Education

Science, Technology, Engineering, and Mathematics (STEM) originated in the United States, emphasizing interdisciplinary integration of the above four disciplines to address real-life problems or projects. With the rapid development of STEM education, the call for increasing humanities and art education in society is getting stronger and stronger. In 2006, STEM added art and formed STEAM education, which emphasized the cultivation of all-around development people with creativity and innovation spirit for future inventors and creators (Connor et al., 2015). STEAM education emphasizes the essential role of individual students in learning to stimulate individual curiosity and effectively promote students to go deep into scientific inquiry.

Science, Technology, Engineering, Arts, and Mathematics education takes the student as the center and cultivates students’ ability to solve problems and innovate. In the teaching process, teachers tell students how to do it and guide students to experience the process of solving practical problems and improve students’ creativity level in exploration (Zhao and Lu, 2016).

There are four standard teaching methods in STEM or STEAM education: problem-based learning, inquiry-based learning, design-based learning, and project-based learning (PBL). Among them, the role of PBL in STEM/STEAM education has been widely concerned. PBL is an approach for students to construct knowledge through teamwork and problem-solving using various scientific methods (Krajcik et al., 1999). This approach pays special attention to students’ awareness of “learning by doing.” Mustafa et al. (2016) made a meta-analysis of STEM in the first decade of the 21st century. They found that PBL was the learning model with the most significant number of integrated STEM curriculum. The meta-analysis results also revealed that an integrative approach could be expanded for students’ development in motivation, interest, achievement, performance, attitude, and perception if the integrated STEM is implemented at the school and higher education levels.

### STEAM/STEM Education and Creativity

Many studies have examined the relationship between STEAM/STEM education and creativity and found a positive association between the two using elementary school to college participants. Some studies focused on STEM-related learning in higher education and examined how STEAM/STEM education influences creativity in higher education. For example, Kuo et al. (2018) applied a STEM Interdisciplinary PBL approach to teaching 45 university students to develop a human-computer interaction (HCI) system to solve real-world problems.

Several studies explored how STEM education could affect student creativity before college. For example, using a single group pre-and post-test design, Lou et al. (2017) examined how a STEM-PBL teaching program affects creativity among ninth graders. Their results supported the program’s effectiveness in promoting students’ creative personalities, such as adventurousness, curiosity, imagination, and accepting challenges.

Similarly, Ozkan and Topsakal (2021) conducted a quasi-experiment to compare two different teaching approaches on students’ learning outcomes. In one session, two seventh-grade classes learned about the concept of “power” in physics. The instructor did not directly teach the idea in the experimental class and asked the students to brainstorm possible solutions. The role the teacher played in the experimental class was scaffolding and facilitator. Students were encouraged to study the phenomenon and came up with their answers. The results demonstrated compared to the control class, students from the STEAM class showed a significant improvement in their creative thinking, measured *via* both verbal and nonverbal forms of the TTCT. In comparison, the teacher in the control class taught the concept directly following the traditional model. Students from the control class were recipients of knowledge rather than explorers of problems.

Some studies conducted direct observation and interviews with students and teachers to examine the benefit of STAEM education on creativity. For example, He et al. (2019) studied how a STAEM teaching model positively influenced middle school students’ creativity. Unlike previous studies, students in this study were asked to make an art project, pottery, through self-exploration and teacher’s scaffolding. In addition, to have experts evaluate students’ creative products, teachers were also interviewed and asked to assess students’ creations in originality and appropriateness.

Case studies provide more in-depth information about how and why STEAM education is beneficial to students’ creativity. In a quasi-experiment, Hathcock et al. (2015) randomly assigned eight ninth-graders into two groups: the treatment and control groups. They asked them to design a buoy that could hold as many golf balls as possible using any materials provided. The teacher adopted an inquiry-based approach in the treatment group, asking questions about their buoys to encourage students to think and discuss more ideas with their partners and peers. In the control group, students were given strategies to solve the program. The study found that students from the experimental group outperformed on measures of creativity than those from the control group. They also interviewed their teachers and concluded that teachers’ guidance and encouragement play a vital role. Teachers’ guidance and support can effectively facilitate students’ creativity than simply asking students to self-explore ways to form innovative products. Moreover, a cooperative learning model also promotes communication among students, thus promoting the solution of problems. Both teachers’ support and students’ cooperation are essential elements of STEAM and STEM education.

Science, Technology, Engineering, Arts, and Mathematics education at the elementary school level also effectively promotes students’ learning outcomes, including creativity. For example, Oh et al. (2013) developed a STEAM Education Program in a sixth grades science class and tested its influence on students’ creativity. This study adopted a Scratch-based STEAM education program for an experimental class of Korean students, whereas students from the control class adopted the traditional learning method. They found that Scratch-based STEAM education had a positive effect on the improvement of creativity.

Siew and Ambo (2018) had two classes of fifth-grade students, one received PBL-STEM learning, and the other received a conventional teaching format. The only difference between the two classes is that the experimental class encouraged students to self-explore but used a more traditional approach. They used the Scientific Creativity Test (SCT) to assess scientific creativity. The results show that the students’ creativity in the experimental group has been improved more than those from the control group.

To sum up, cumulative evidence has demonstrated a positive influence of STEAM/STEM education on creativity. We want to highlight three essential elements in these educational programs: cooperative learning among students, teacher guidance and support, and PBL. We also observed some limitations of these studies. First, the measurement for creativity was primarily based on a narrow definition of divergent thinking, and as a result, scores on TTCT were the only indicator for creativity. As discussed earlier, creativity is a multilevel and multi-facet concept, one measurement cannot fully capture the essence of creativity. Second, although STEAM/STEM education adopted an integrated approach across different subject areas, most studies focused on just one or two subject areas, such as technology or science. Third, many studies adopted a single-group pre/post experimental design and did not have a control group, which cannot rule out the influence of many confounding variables. Lastly, we noticed that some studies have adopted the PBL approach and have students completed a final project as part of the learning curriculum. Unfortunately, the final product was rarely used as an indicator of creativity. We believe the final projects often involved creative thinking and should be assessed to examine creativity.

### Current Investigation

Based on the results of previous studies, we propose a new PBL study, in which we integrated five subject areas of STEAM *via* a fourth-grade science class. The five subject areas include science, technology, engineering, art, and mathematics.

The purpose of this study is to compare a new PBL STEAM educational program to a conventional science educational program to see the effectiveness of their learning outcome. We also compared their creativity at both individual and team levels. We took a multi-method approach to assess students’ creativity, including an assessment of divergent thinking, a project-orientated measure *via* the CAT, and a measurement of CSE. Additionally, we assess students’ creativity at the group level by having experts evaluate the final group product.

We chose two classes and randomly selected one as the experimental group and the other as the control group. The two classes are comparable in their academic preparation. We started the study at the beginning of a fall semester, which they began a new unit in a science class. The same instructor taught the two groups at the same time, with the same objective. Additionally, we gave the two groups the same creativity measures before and after the intervention. The only difference between the two groups is the teaching approach, detailed in the Materials and Methods section.

There are two major hypotheses. First, we hypothesize a multi-approach measurement of creativity can effectively capture creativity; therefore, there is a consistency among all measurements of creativity. The scores on these measurements positively correlated to each other. Second, we hypothesize that students from the experimental group improve more than those from the control group on creativity after the intervention.

## Materials and Methods

### Participants

Participants were 68 fourth graders (40 male, 28 female) from two natural classes in a southern city, China. The two classes were comparable in terms of student academic preparation and students’ performance in science education. Among the 68 participants, two students did not complete the measurement of creativity and were excluded from the sample. For 6 weeks, one of the two classes was randomly selected as the experimental group (*n* = 33, 12 female) to receive a PBL STEAM program. In contrast, the other class was selected as the control group (*n* = 33, 14 female), in which students received a traditional science class at the same time. A female science teacher served as the instructor for both classes.

### Measurements

All students received creativity measurements before and after the 6-week classes. During the intervention, students worked in groups of four members to produce a musical instrument, which was rated twice by experts for creativity, one at an earlier design and the other when the product is completed In addition, they also completed a test on scientific achievement after the intervention. The measurements include the following.

#### Creativity Measurements

We used a multi-method approach to examine student creativity dynamically at both individual and team levels. It includes a test of divergent thinking, a story completion using the consensus assessment technology, and a self-report on self-efficacy. In addition, we asked the students from the two classes to complete a creative product in teams. Details for each assessment are introduced as the following:

##### Test of Divergent Thinking

Based on Guilford’s divergent thinking theory, the test asked participants to generate as many unusual uses for an ordinary object as possible such as “paperclip” (in pre-test) and “match” (in post-test) as contents of tests. We only calculate the fluency score on this task, the number of different ideas a person generated in a specific time. Two graduate students counted independently, and their agreement was above 0.90.

##### Story Completion

We gave students a word prompt and asked students to complete a story based on a prompt word, which was “keyhole” (in the pre-test) or “robot” (in the post-test). Using postgraduate students to serve as expert judges for creativity has been used extensively in creativity literature (Kaufman et al., 2008). Using the CAT (Amabile, 1982), three graduate students with at least 1 year of experience studying creativity served as expert judges. They each independently rated all stories based on their subjective criteria, providing a rating on originality, using a seven-point scale, with a “1” representing least original and “7” representing most original. The inter-rater reliability of the experts was above 0.85. We calculated the average scores of the three experts’ ratings to represent a student’s originality on this task.

##### The Idea Evaluation Self-Efficacy Measure

It was developed by Steele et al. (2018) to measure CSE by having participants rate the level of confidence toward their abilities to evaluate new ideas using a five-point Likert scale. A sample item is, “When evaluating new ideas, I can quickly and accurately determine if it will be successful.” The survey has established an acceptable internal consistency (*α* = 0.76).

##### Creative Products

Throughout the 6-week intervention, students worked in groups of four members and were asked to create a blueprint for a musical instrument, construct the instrument, and then perform a piece of music using the instrument. Two specific creative products were evaluated: the blueprint and the performance of the musical instrument (a video clip). Similar to the procedure for assessing the story completion tasks, we invited three graduate students to independently rate the products on originality and appropriateness. They were asked to use a seven-point scale, with a “1” representing least original (or appropriate) and “7” representing most original (or appropriate). Each group of participants obtained two originality and two appropriateness scores, one for the blueprint, and the other for the final product. The inter-rater reliability of the three experts was above 0.80. We calculated the average scores of the three judges to represent originality and appropriateness scores at the team level.

#### Scientific Achievement Test

Under the current educational environment in China, many teachers are hesitant to carry out STEAM education because they are worried that STEAM education will negatively affect students’ learning and academic achievements. To investigate whether STEAM education will affect students’ academic achievements, we also require all students to complete the scientific achievement test in the school district.

### Experimental Design, Curriculum, and Teaching Approach

The study adopted a quasi-experiment with a pre-test/post-test. There were two groups, the experimental group, and the control group. The same teacher taught the two groups based on the same science curriculum in Zhejiang Province. Students from the two groups had the same learning objective: understanding the sciences of music and sound. In 6 weeks, students learned about different subject areas relating to music and sound. These areas are (1) physics, learning about the mechanism of vibration and sound waves; (2) engineering, understanding how to construct objects with different pitches of sound; (3) mathematics, measuring in pitch, volume, tempo, and rhythm; (4) music, appreciating tunes, pitches, tempo, and rhythm; and (5) art, drawing a design for an instrument. As a part of the class evaluation, students worked in groups to design and construct a musical instrument, which they would play at the end of the study unit.

The two groups differed in teaching approaches. In the experimental group, the teacher adopted a PBL based STEAM program. Using an interdisciplinary approach, the teacher taught the knowledge of the five subjective areas in an integrated fashion, assisting students to complete a music instrument. Therefore, the course was project-based and student-orientated. Students were asked to work in groups to engage in hands-on learning from the beginning to design and construct a musical instrument. They were encouraged to self-explore and problems-solve using various knowledge (i.e., science, technology, engineering, arts, and mathematics).

On the contrary, the teacher used a conventional teaching approach to teach the same content in the control group. Different from the experimental class, the teacher adopted the traditional science teaching approach when delivering the same content across five different subject areas to the control class. The primary teaching mode was lecturing, and only toward the end did the teacher ask students working in groups to incorporate the knowledge learned in previous lectures to make a musical instrument.

#### The Curriculum of Experimental Group

In the experimental group, students received a project-based STEAM program to create a ukulele. Students were asked to integrate knowledge from different subject areas in STEAM and work to produce their creative products.

The program included six different sessions based on the principle contents of the Chinese national standard of science education. The teaching objective was to make a ukulele and to play a piece of music. There are five objectives in learning about science, technology, engineering, arts, and mathematics. The scientific aim is to have students understand the sound principle, recognize a ukulele’s vocal principle, and observe, compare, and analyze the ukuleles made of different materials. The technical goals are using networks to collect useful information, using various tools to make the Ukulele, designing the Ukulele according to requirements, and drawing a diagram to make it visible and operable. The engineered objectives are selecting appropriate materials to make a ukulele, producing a ukulele according to blueprint, testing, and adjusting during and after the production process, and evaluating others’ designed products, and making suggestions. The art objectives are to design and decorate the Ukulele. The mathematical objectives are calculating the project cost and measuring the length of the string accurately. Each subject has specific objectives for students, while all program sessions focus on reaching the final goal: to make ukuleles as groups and play them. The program includes six sessions, which are described below.

Session 1 involved an introduction of the Ukulele, knowing the history, structure of the Ukulele, and learning about the vocal principle of the Ukulele. Moreover, the students were asked to disassemble the Ukulele to understand the system and principles of the Ukulele and comparing different materials to choose the most suitable one as the strings. And create a real scene for them, telling them that they need to design and make Ukulele for the factory.

In Session 2, students were to design a ukulele and draw the blueprint through group cooperation. Session 3 allowed students to have opportunities to make the Ukulele according to the schematic diagram. Through discussion, the teacher leads students to think about the possible problems through the making process.

In Session 4, students were to decorate the Ukulele. The teacher and members of the groups can evaluate the quality of the products and team cooperation through the program.

In Session 5, students made a four-string ukulele in groups and marked the syllables. After that, they were asked to play the Ukulele they made and discuss the advantages and disadvantages of the products and make progress in them.

Students’ products, including blueprints and performance of musical instruments, were collected to evaluate students’ creativity during the STEAM program. In Session 6, students indicate the price of the product and explain the product. After that, both the teacher and students evaluate the creative product and assess the participating situation throughout the whole project. Teachers also presented a review of all lessons and asked students to make improvements to their Ukulele.

#### The Curriculum of Control Group

In the control group, students received regular science classes with objectives on mastering sound and core concepts and were evaluated by homework, class performance, and tests.

The teacher used a standardized textbook as the primary resource for the science class. The class also includes six sessions.

In Session 1, students were asked to describe the sound around them and introduce the principle of making sounds. In Session 2, the teacher using different materials to help students understand how the sound travels. Session 3 allowed students to know about the auditory sense through models. In Sessions 4 and 5, students learned about the relationship between amplitude and loudness, the vibration frequency, and sound level separately. In Session 6, students were asked to make instruments in groups.

#### Intervention Process

Before conducting the study, we obtained approval from an ethical review committee at the host university in Beijing and a letter from the collaborative school (a public school in southern China) to meet the ethnic standard. The school has allowed the intervention program to integrate into the existing academic curricula as a part of their educational reform in science education. Both students and their parents received a welcome letter explaining the purpose and procedure of the study. Parents’ consent and student assent were obtained.

The study took place in 8 weeks in the fall semester of 2020. The first and the last week were to assess students’ outcomes. The 6 weeks in the middle were used for intervention, in which students received a 45-min class each week. A female teacher with a bachelor’s degree in science education and 2 years of science teaching experience taught both groups. Details of the program and different teaching approaches are listed in Table 1.

**Table 1:** Main contents of sessions in the experimental group and the control group.

Sessions	Contents		Subjects	
	Experimental	Control	Experimental	Control
Pre-test	Divergent thinking, story completion, and self-efficacy			
Session1	Sound creation, Sound perception, and Instruments	Sound creation and Sound perception,	Sciences Art	Sciences
Session2	Instrument design	Transmission of sound	Sciences, Technology Engineering Art	Sciences
Session3	Material selection, Instrument creation	know about the auditory sense through models	Sciences, Technology Engineering Art Mathematics	Sciences
Session4	Sound volume, Sound pitch, and instrument-music modification	relationship between amplitude and loudness, the vibration frequency, and sound level separately	Sciences, Technology Engineering	Sciences
Session5	Instrument and Sound instrument play		Sciences and Engineering	
Session6	Instrument appearance, Instrument price, and Instrument’s instruction	Instrument’s creation	Sciences, Technology Engineering Art Mathematics	Sciences Technology Engineering Art Mathematics
Post-test	Divergent thinking, story completion, and self-efficacy			

Compared with the control group, the experimental group has the following characteristics: Firstly, emphasis on multidisciplinary integration, requiring students to apply knowledge to solve problems interdisciplinary; Secondly, teachers have adopted the PBL teaching method, highlighting the concept of “learning by doing” in the teaching process, guiding students to explore with the goal of product production, and giving appropriate support to students when exploring; Thirdly, students solve problems through group cooperation in each lesson. However, the control group did not emphasize subject integration; teachers did not adopt the PBL teaching method; students learn scientific knowledge in the first five sessions and were asked to work together to produce a product in the last session.

## Results

### Creativity Across Different Measurements

We adopted a Pearson correlation analysis to examine the first hypothesis regarding the consistency across all three measurements for individual creativity: the divergent thinking task, the story completion task, and the CSE measurement. The results showed that all three individual creativity scores (fluency, originality, and CSE) are moderately correlated (*r*s are 0.47, 0.22, and 0.13, respectively). Principle Analysis supported a one-factor model, explaining 52.65% of the total variance. All three variables loaded on the factor at above 0.5, suggesting although the three measurements may capture different aspects of creativity, they are also consistent in capturing one principal component.

### The Impact of Teaching Methods on Creativity

#### Domain-Specific Knowledge Through Test of Academic Achievement

The school district provides academic tests for all students who participate in new knowledge about “sound” 6 weeks. An independent-sample *t*-test was applied to examine the difference between the two groups on their academic achievement. The results showed no difference between the two groups (*t* = −1.16, *p* > 0.05).

These results reflect the fact that students from both groups have accomplished the unit objective in the fourth-grade science education, which is to understand the science of music and sound.

#### Impact of STEAM Education on Individual Creativity

**Figure 1:**
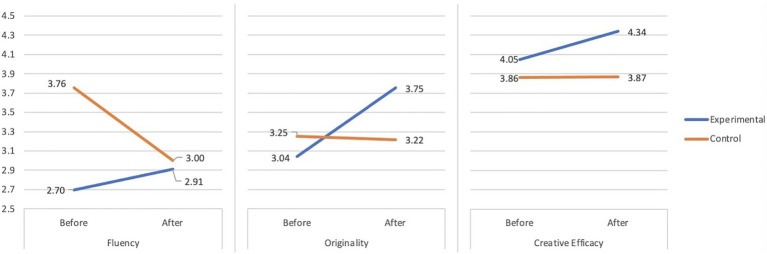
The improvement of individual creativity in the experimental group and the control group.

Figure 1 showed that after a 6-week intervention, students from the experimental group improved significantly on overall ratings of creativity across different tasks (fluency score on divergent thinking test, originality score on story completion, and CSE). In contrast, the control group showed no improvement.

To examine the effectiveness of the STEAM program in the improvement of students’ creativity, a 2 (Group: Experimental vs. Control) × 3 (creativity: fluency, originality, and CSE) × 2 (time: before vs. after) mixed design ANCOVA was conducted with “group” as a between-subject variable, and “creativity” and “time” as within-subject variables, and the academic achievement as covariate variable.

The results showed a marginally significant interaction between time and group [*F*_(1,64)_ = 3.286, *p* = 0.075, *η_p_*^2^ = 0.050]; this result supported the second hypothesis that students from the experimental group improved more on creativity than those from the control group (Table 2).

**Table 2:** The statistical results of ANCOVA.

Variables	*df*	*MSE*	*F*	*p*	η*_p_*^2^
Group	1	0.093	0.015	0.902	0.000
Time	1	0.842	0.277	0.601	0.004
Time × Group	1	10.009	3.286^+^	0.075	0.050
Academic Achievement	1	15.385	15.385	0.119	0.038

+ *p* < 0.1.

#### Impact of STEAM Education on Creative Projects (Team-Level)

To examine the effects of the intervention on team-level creativity over intervention, we conducted two multilevel nested model analyses, one for each of the two difference scores, namely, the difference between blueprints and final products on team originality and team appropriateness. For each analysis, two independent variables were selected, which were (1) changes in individual originality (i.e., differences in expert ratings on story completion between pre-and post-test) and (2) group (i.e., experimental vs. control). The results showed a significant main effect for the group on team originality (*t* = 3.573, *p* < 0.05), which indicated that the experimental group improved (mean difference = 0.99) more than the control group (mean difference = −1.29). Moreover, we also found a significant main effect for time (*t* = −2.306, *p* < 0.05). The changes of individual originality from time 1 (mean = 3.04, *SD* = 0.89) to time 2 (mean = 3.75, *SD* = 1.25) from the experiment group had a significant impact on team originality; however, no such effect was found in the control group.

The results support our second major hypothesis, which states that students from the experimental group improved more than those from the control group on creativity after the intervention.

**Figure 2:**
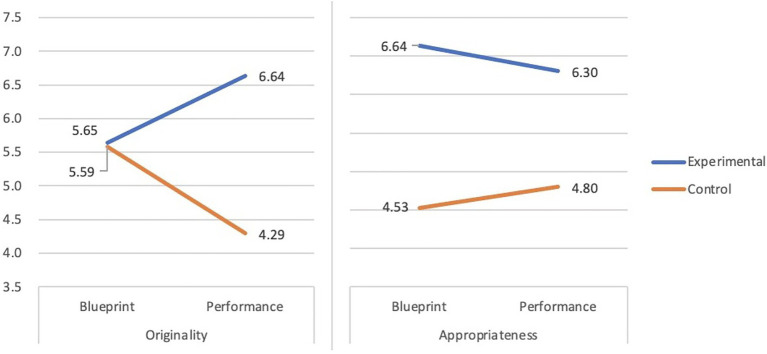
The improvement of team-level creativity in the experimental group and the control group.

**Figure 3:**
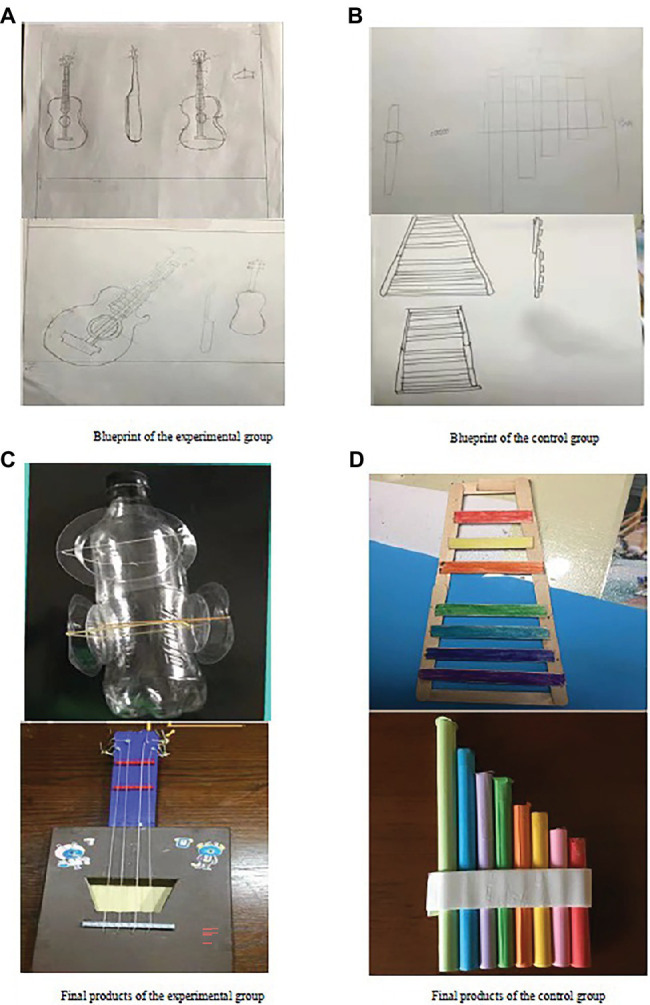
Creative products (blueprint and the pictures of final products) from the experimental group and the control group.

Figures 2, 3 further illustrate the difference between the two groups in terms of blueprint and final product (the performance of the musical instrument).

From the above figures, the differences in blueprints between the two groups may not be noticeable in terms of originality (see Figures 3A,B). However, the final products from the experimental group showed more novelty in both material selection and production delivery (Figure 3C) than those from the control group (Figure 3D). We should also mention that we recorded students’ performance and found that the quality of the sound generated by the instruments from the experiment group closely resembled the actual instrument than those from the control group.

Two primary reasons might be attributable to the overall advanced performance on creativity from the experimental group than the control group. First, when teaching the experimental group, the teacher consciously applied an interdisciplinary approach and encouraged students to use the approach to solve problems. This approach allowed students to think holistically and engaged in more divergent thinking. In contrast, when teaching in the control group, the teacher primarily adopted a dedicative approach to delivering basic knowledge of the science of music and sound. As a result, she provided students with less opportunity to self-explore.

Second, by using the PBL approach, students from the experimental group were involved in more hands-on activities than the control group. The learning objective of creating a musical instrument was made clear from the very beginning. In contrast, students from the control group spent a significant amount of time learning knowledge in different disciplines related to the production of the instrument. Yet, they only engaged in a hands-on learning experience toward the end of the unit study. As a result, students from the experimental group seemed to have a higher level of intrinsic motivation, which might lead to more original products than the control group.

## Discussion

In this study, we used a quasi-experimental pre-test/post-test design to explore the effectiveness of a project-based STEAM program in improving student creativity. After a 6-week intervention, we measured the creativity level of the two groups of students by various methods to compare the effects of two different teaching methods. We found that diversified creativity measurement methods measure students’ creativity effectively. Students in the experimental group who received the PBL STEAM program improved creativity at both individual and team levels.

One significant feature of this study is to use a multi-method to measure students’ creativity. Compared with previous studies, we measure students’ creativity at the individual level and reflect students’ creativity at two levels: the little-c and the mini-c. At the same time, Pearson correlation results show that all the scores for measuring individual creativity are positively correlated. And they can be loaded in one factor. That is to say, all the measures of creativity used in this study are consistent. This verifies our first hypothesis (A multi-approach measurement to creativity can effectively capture creativity; therefore, there is a consistency among all measurements of creativity. The scores on these measurements positively correlated to each other). In addition, we also test whether there is consistency among the various introductions of creativity. Our research measures and analyzes creativity from different angles and levels, avoiding the one-sidedness of a one-dimensional test and making our results more convincing.

Our results showed no difference between the two groups in tests of science achievement. Such a result reflects that students from both groups have the same level of mastery in understanding the science of music and sound. It is important to note that although students from the experimental group spent less time learning specific topics in science education than the control group, they gained more time on hands-on experience, which may help them gain a deeper understanding of the concept. More importantly, through PBL, students from the experimental group may have a higher level of intrinsic motivation toward the science, and as a consequence, enhance their STEAM-related creativity. These results demonstrate the primary benefit of STEAM education on student learning outcomes may not depend on their academic achievement but creativity.

The fact that the creativity level of the experimental group is higher than that of the control group at both the individual level and the group level verifies our second hypothesis, which states that students from the experimental group improved more than those from the control group creativity after the intervention. Our results are consistent with the research results of Oh et al. (2013); Siew and Ambo (2018); and He et al. (2019).

One unique feature of this study is to measure team creativity in addition to individual creativity. Our results regarding the significant improvement in team creativity from the experimental group provided additional evidence to the benefit of PBL based STEAM education in improving both individual and team creativity while ensuring the quality of knowledge learning. We suspected that the improvement of the creativity level of the experimental group might be due to the following reasons.

First, the teacher in the experimental condition adopted a multi-discipline and integrated approach to teaching science. In the teaching process, the teacher encouraged students to integrate knowledge across different disciplines to solve practical problems. Interdisciplinary or interdisciplinary knowledge can often provide learners with a variety of problem-solving methods, broaden their thinking, and enable individuals to break through the stereotype caused by specific domain knowledge and solve problems creatively. This transcendence reflects the originality, fluency, and flexibility of thinking (Zhang and Gu, 2004). However, in the control condition, the teacher used a more conventional approach, in which students learn different units of science sequentially. For example, unlike teaching in the control condition, the teacher integrated different subjective goals in the experimental condition, that every session included different goals from STEAM subjects. For example, in Session 2, students from the experimental group were asked to work designing a ukulele and drawing a blueprint that later is completed. The creation process of a ukulele involves the knowledge of science, technology, engineering, and arts. With direct guidance from the teacher, students were reinforced with the concept of integration of various disciplines.

The second reason to explain why having the 6-week intervention can be effective, using the PBL teaching method; students were clear what the end product would be. Students were consistently reinforced to use multidisciplinary knowledge to solve the problems, with a great emphasis on “learning by doing.” This feature allowed students to have more chances to take self-explored learning. This stimulated students’ interest and promoted their intrinsic motivation, subsequently encouraging students’ creativity (Kuo et al., 2018). For example, students in the experimental group were more eager to express their ideas and raised their hands to answer the questions more often. And in the process of inquiry, students have been given more specific guidance from the instructor. For example, in making a Ukulele, if students encounter difficulties and cannot make musical instruments sound, teachers will lead and encourage students to find out the problems and provide some ideas to solve them. Therefore, with teachers’ help and encouragement, students were able to work more effectively and creatively in creating the music instrument throughout the invention process. This result supports a claim that explicit motivation can also facilitate Chinese students’ creativity (Kaufman and Niu, 2012). It is also consistent with findings from a previous study in which Chinese students produce more creative products under a more elaborated instruction on how to be creative than a mere encouragement of creative expression and a control condition without mentioning creativity (Niu and Liu, 2009).

Lastly, students in the experimental condition also began working in groups of four from the first week of intervention to brainstorm ideas and collaborate in learning, which allowed more collaboration among the team members. In contrast, students in the control condition learned various contents by listening to teachers’ lectures and demonstrations. Although students from the control group also had an opportunity to form a group and complete the same assignment as their counterparts in the experimental group, the group formation took place in Session 6 of the intervention in the control condition, rather than in all sessions in the experimental condition. Through the interaction among group members, students in the experimental group were allowed to exchange their ideas and communicate to gain deeper learning, which positively impacted their creativity. The previous research also showed that through the cooperation of different subjects, students’ creativity could be improved after the STEAM program (Siew and Ambo, 2018), which is consistent with our study.

## Limitation and Future Research

The study has some limitations. The first one is the task itself. Students only engage in one project during the intervention period: the design and creation of a musical instrument. Future studies should design STEAM programs that allowed students to create different instruments and implement them to examine the effect. The second limitation is the presence of the experimenter effect. We invited one teacher to teach both experimental and control groups. Although the instructor realized a comparison between the two groups, the instructor tried to exhibit the same level of enthusiasm. She may unconsciously bring her own bias into the study. Because of the easy implementation, the teacher used Ukulele as an example. Future research should recruit two teachers with comparable teaching experience and styles to implement STEAM programs and groups.

This study offers two important implications. First, our study demonstrates that using a multi-method approach to measuring creativity is a better way to capture student creativity in a broad sense. This can also help educators see the level of changes in creativity throughout the intervention to be more aware of creativity as an essential learning outcome. Second, an important observation from this study is that a PBL based education not only can have a direct benefit to students, but it may also have a direct impact on teachers. Educators are more willing to encourage students to think divergently and express themselves more, positively influencing student creativity. Previous studies have shown that creativity is not a trait that educators are particularly interested in promoting (Zhang, 2009). We believe the STEAM program like the one described in this study will long-term impact students’ learning outcomes, especially promoting their creativity.

## Conclusion

In conclusion, this study contributes to creative research by using a multi-method approach to measure creativity. It also demonstrates that a PBL and an integrative, multidisciplinary approach in science education can improve students’ creativity, which provides practical insights in promoting creativity in education in general.

## Data Availability Statement

The raw data supporting the conclusions of this article will be made available by the authors, without undue reservation.

## Ethics Statement

The studies involving human participants were reviewed and approved by The Research Ethics Review Board at Beijing Normal University (ethical review number BNU202106100010). Written informed consent to participate in this study was provided by the participants’ legal guardian/next of kin.

## Author Contributions

All authors contributed to the study. LC and WN: conceptualization, design, and methodology. LC, WN, MH, and YZ: formal analysis and experimental operation. MW and YC: writing – original draft preparation. LC and WN: writing – review and editing. LC: funding acquisition. LC and YZ: resources. LC and WN: supervision. All authors contributed to the article and approved the submitted version.

## Funding

This work was supported by the International Joint Research Project of the Faculty of Education, Beijing Normal University (ICER201904).

## Conflict of Interest

The authors declare that the research was conducted in the absence of any commercial or financial relationships that could be construed as a potential conflict of interest.

## Publisher’s Note

All claims expressed in this article are solely those of the authors and do not necessarily represent those of their affiliated organizations, or those of the publisher, the editors and the reviewers. Any product that may be evaluated in this article, or claim that may be made by its manufacturer, is not guaranteed or endorsed by the publisher.
